# What Drives the Occurrence of the Melioidosis Bacterium *Burkholderia pseudomallei* in Domestic Gardens?

**DOI:** 10.1371/journal.pntd.0003635

**Published:** 2015-03-24

**Authors:** Mirjam Kaestli, Glenda Harrington, Mark Mayo, Mark D. Chatfield, Ian Harrington, Audrey Hill, Niels Munksgaard, Karen Gibb, Bart J. Currie

**Affiliations:** 1 Global and Tropical Health Division, Menzies School of Health Research, Darwin, Northern Territory, Australia; 2 Research Institute for the Environment and Livelihoods, Charles Darwin University, Darwin, Northern Territory, Australia; University of California San Diego School of Medicine, UNITED STATES

## Abstract

Melioidosis is an often fatal infectious disease affecting humans and animals in tropical regions and is caused by the saprophytic environmental bacterium *Burkholderia pseudomallei*. Domestic gardens are not only a common source of exposure to soil and thus to *B*. *pseudomallei*, but they also have been found to contain more *B*. *pseudomallei* than other environments. In this study we addressed whether anthropogenic manipulations common to gardens such as irrigation or fertilizers change the occurrence of *B*. *pseudomallei*. We conducted a soil microcosm experiment with a range of fertilizers and soil types as well as a longitudinal interventional study over three years on an experimental fertilized field site in an area naturally positive for *B*. *pseudomallei*. Irrigation was the only consistent treatment to increase *B*. *pseudomallei* occurrence over time. The effects of fertilizers upon these bacteria depended on soil texture, physicochemical soil properties and biotic factors. Nitrates and urea increased *B*. *pseudomallei* load in sand while phosphates had a positive effect in clay. The high buffering and cation exchange capacities of organic material found in a commercial potting mix led to a marked increase in soil salinity with no survival of *B*. *pseudomallei* after four weeks in the potting mix sampled. Imported grasses were also associated with *B*. *pseudomallei* occurrence in a multivariate model. With increasing population density in endemic areas these findings inform the identification of areas in the anthropogenic environment with increased risk of exposure to *B*. *pseudomallei*.

## Author Summary

Melioidosis cases are on the rise in endemic areas of northern Australia and Thailand. This potentially severe infectious disease affecting humans and animals in the tropical belt is caused by the gram negative bacterium *Burkholderia pseudomallei*. Domestic gardens are a common point of exposure to these environmental bacteria and *B*. *pseudomallei* are more prevalent in the dry season in gardens when compared to other areas. This is why we analysed whether common gardening practices such as regular watering (irrigation) or soil fertilizing change the occurrence of *B*. *pseudomallei*. We conducted a soil microcosm experiment with a range of fertilizers and soil types as well as a longitudinal interventional study over three years on an experimental fertilized field site in an area naturally positive for *B*. *pseudomallei*. Irrigation was the only consistent treatment to increase *B*. *pseudomallei* occurrence over time. The effects of fertilizers upon these bacteria depended on soil texture, physicochemical properties such as pH or salinity and vegetation. *B*. *pseudomallei* occurrence was also associated with imported grasses. With increasing populations in endemic areas, these findings inform the identification of areas in the anthropogenic environment with increased risk of exposure to *B*. *pseudomallei*.

## Introduction

Southeast Asia and tropical Australia have recently experienced a surge in melioidosis, an often fatal infectious disease caused by the saprophytic environmental bacterium *Burkholderia pseudomallei* [1,2]. Case numbers in the Top End of Australia have substantially increased in recent years. In the 20 years from 1989 until 2009 there was a median of 27 cases annually [3]. In the last 5 years there has been a median of 64 cases annually and in each of two recent years, 1 in every 2,000 people living in the Top End has had culture confirmed melioidosis [4]. *B*. *pseudomallei* are found in soil and water world-wide in the tropical belt with the major endemic region being southeast Asia and tropical Australia [5–10]. *B*. *pseudomallei* is an opportunistic pathogen able to infect humans [11] and a large variety of animals [12]. Humans with a compromised immune system such as from diabetes, hazardous alcohol use, chronic renal disease and immunosuppressive therapy are at particular risk of acquiring and dying from melioidosis [13]. Clinical presentations vary widely and include skin and soft tissue abscesses, pneumonia and disseminated infection with septic shock, the latter having mortality rates above 80% [14].

The Darwin area (12° S latitude) in the tropical north of Australia is endemic for melioidosis and gardening is considered to be an important recreational and occupational source of exposure to and ultimately, infection with *B*. *pseudomallei* [3]. In the 20-year Darwin prospective melioidosis study, 407 (75%) of 540 consecutive melioidosis patients had documented recreational activities such as gardening or outdoor sporting activities where exposure to *B*. *pseudomallei* was considered likely to occur [3]. Domestic gardens are not only a common ground for humans to be exposed to the environment, but *B*. *pseudomallei* might also thrive in the garden habitat. While *B*. *pseudomallei* and melioidosis predominate in the monsoonal wet season [3], previous work in rural Darwin found that in the dry season *B*. *pseudomallei* is more often present in domestic gardens than in farms or environmentally less disturbed areas [15]. This might be attributed to the widespread use of irrigation during the dry season. Being a non-spore forming, gram negative bacterium, *B*. *pseudomallei* is often, but not exclusively associated with moist soil close to a water source and with surface water or alluvial areas as well as rice fields [7,15–19]. At environmentally disturbed sites, *B*. *pseudomallei* was associated with pens or paddocks for pigs, chickens or horses with an average odds ratio of 3.8 [15]. This raises the possibility that soil aeration through digging activities or organic material and nitrogen from animal waste support growth of *B*. *pseudomallei* [15].

In this study, we addressed the hypothesis that anthropogenic manipulations associated with gardens such as the use of irrigation, fertilizers, commercial potting mix or keeping pets influence the habitat of *B*. *pseudomallei* and change its abundance and/or occurrence. We conducted a soil microcosm experiment with a selection of fertilizers as well as a longitudinal study over three years on an experimental fertilized field site in a location naturally endemic for *B*. *pseudomallei*.

## Materials and Methods

### Experimental field site

**Fig 1:**
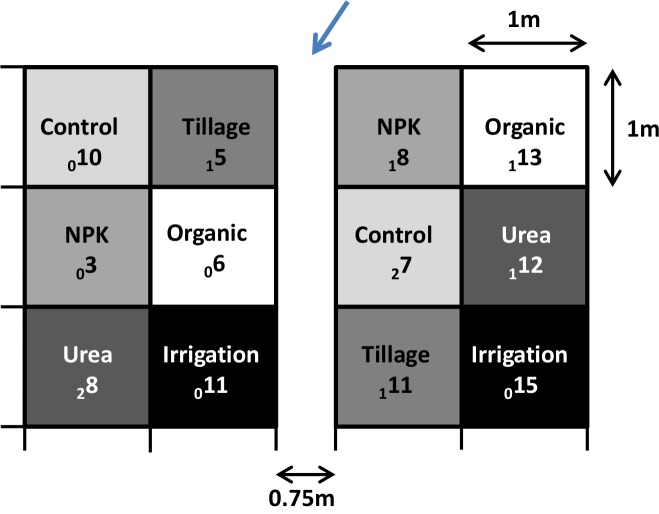
The experimental field site. The setup consisted of two plots with six quadrants each (1x1 metre) with type of treatments or control indicated. Soil of two holes per quadrant (different holes for each round) was tested for *B*. *pseudomallei* on a quarterly basis approximately. The arrow marks the direction of the water run-off in the wet season. The number indicates the *B*. *pseudomallei* occurrence with the subscript number referring to the baseline occurrence before start of treatment (total 2 holes tested per quadrant) followed by the number of *B*. *pseudomallei* positive holes during treatment (total 26 holes tested per quadrant for the duration of the experiment).

In August 2008 an experimental site was established on a private property in rural Darwin in an area that previously tested positive for *B*. *pseudomallei*. The soil at this site was a hydrosol [20] and the soil texture of the topsoil was clay with a subsoil consisting of grey clays and siltstone. The site consisted of two plots, 0.75 metres apart and each plot had six 1x1 metre quadrants (Fig. 1), which included a control quadrant and five quadrants with different treatments which represent common garden practices in the Darwin region (Table 1). Treatments were applied every two weeks with water application every 2^nd^ day for three years. Timing and dose reflected local garden practices.

**Table 1:** Treatments applied to the experimental field site over three years.

Treatment	Details
Tillage	Two-weekly turnover of soil
Irrigation	Every 2^nd^ day, application of 20L of unchlorinated water
Organic	Two-weekly application of organic fertilizer with 20L of water (Yates, Australia; composted chicken manure, blood and bone, fish meal and seaweed; N 3.7%, P total 2.0%, K 1.8%)
NPK	Two-weekly application of NPK fertilizer with 20L of water (Tropigro, Australia; N from ammonium sulphate 10.3%, P total 9.0%, K from potassium chloride 7.0%)
Urea	Two-weekly application of 20L of 1:20 diluted human urine (final urea concentration 0.05%) to mimic the presence of animals in gardens

The water used was unchlorinated water from the property’s bore with a pH of 7.5 containing 50 mg/L calcium carbonate and which repeatedly tested negative for *B*. *pseudomallei* by culture.

There were 14 rounds of soil sampling and in each round 2 random soil samples were collected from each of the 12 quadrants to give a total of 336 soil samples. The first sampling round was before the start of the experiment in August 2008 followed by sampling every two months in year-1, every three months in year-2 and every four months in year-3 of the experiment, with the last round in August 2011. Soil from a depth of 20–30 cm was collected into sterile 50 mL specimen containers and auger and spade were cleaned with 70% ethanol between soil collections [21]. Soil moisture was determined as described previously using the Australian Soil and Land Survey Field Handbook [21,22]. Soil pH was measured using a soil pH field test kit (Inoculo, Australia). In the last 6 months, soil electrical conductivity (EC) was measured using the Field Scout EC Meter (Spectrum Technologies, USA). Grasses covering the experimental field site were identified by the Northern Territory Government Herbarium and were either *Sorghum* spp. (spear grass) or *Pennisetum pedicellatum* (annual “mission grass”). At the time of sampling, the presence or absence of live specimens of these grasses at the sampling hole was noted.

### Microcosm experiments

Of 120 250-mL clean and autoclaved plastic containers, 30 were each filled with either 130 g of commercial “garden soil”, sandy clay loam, clay or sand (Table 2). The non-commercial soil was collected in rural Darwin and tested negative for *B*. *pseudomallei* by culture. Nine different treatments plus controls (no change) were applied in triplicate to the containers (Table 3). Treatments included the addition of distilled water or distilled water in combination with eight fertilizers which are commonly used in residential gardens in the Darwin region. After two weeks of soil conditioning at 32 degrees Celsius in the dark, all soils were inoculated with 5x10e4 CFU of an environmental strain of *B*. *pseudomallei* (MSHR2817) which has the commonly found multi-locus sequence type (ST) 144 [23] and incubated at 32 degrees Celsius for four weeks in the dark. Soil DNA was extracted and *B*. *pseudomallei* DNA detected as described below.

**Table 2:** Microcosm experiment soil types.

	VSW (%)**	pH	EC (μS/cm)
Commercial “garden soil”*	58.0	5.4	700.0
Sand	< 1.0	5.5	12.7
Sandy clay loam	4.9	5.8	8.3
Clay	2.0	5.5	35.6

* composted (>60%) organic-based soil blend with organic garden fertilizer added (NPK 3.4%: 1.5%: 1.3% w/w) (Hortico, Australia)

** volumetric soil water content

**Table 3:** Microcosm experiment treatments.

	Microcosms experiment: nine treatments								
	dH_2_O	Urea	NH_3_	K;NO_3_^-^	P	Organic *	Fe	Mg	CaC0_3_
**Product name**	NA	Thrive all purpose	Acticote natives	Thrive flower & fruit	Orchid Food	Organic slow release, 10–88	Soil acidifier	Epsom salt	Garden lime 32% w/w
**Brand (Australia)**	NA	Yates	Yates	Yates	Yates	Tropigro	Richgro	Richgro	Richgro
**Amount (g) (g for sand)**	**	0.26 (0.15)	0.26 (0.15)	0.26 (0.15)	0.26 (0.15)	1.12(1)	0.26 (0.15)	0.26 (0.15)	0.8
**N tot**	NA	27.0	19.0	15.0	21.5	4.0	NA	NA	NA
**NH**_**3**_	NA	2.6	**10.2**	3.6	5.5	0.1	NA	NA	NA
**NO**_**3**_^**-**^	NA	3.0	5.1	**8.8**	4.5	NA	NA	NA	NA
**Urea**	NA	**21.4**	3.7	2.6	11.5	NA	NA	NA	NA
**P**	NA	5.5	0.3	4.0	**8.3**	3.2	NA	NA	NA
**K**	NA	9.0	9.0	**26.0**	13.0	2.0	NA	NA	NA
**B**	NA	0.005	NA	0.005	0.005	0.001	NA	NA	NA
**Na**	NA	NA	NA	NA	NA	0.420	NA	NA	NA
**Mg**	NA	0.500	0.100	0.500	NA	0.900	NA	**9.0**	NA
**S**	NA	0.200	6.000	NA	0.060	0.500	**11.5**	**13.0**	NA
**Ca**	NA	NA	0.200	NA	NA	12.000	NA	NA	NA
**Mn**	NA	0.040	NA	0.040	0.040	0.060	NA	NA	NA
**Fe**	NA	0.180	NA	0.180	0.006	0.160	**20.0**	NA	NA
**Cu**	NA	0.005	NA	0.005	0.005	0.004	NA	NA	NA
**Zn**	NA	0.020	NA	0.020	0.020	0.080	NA	NA	NA
**Mo**	NA	0.002	NA	0.002	0.002	<0.001	NA	NA	NA

Nine treatments used (plus control) which were each applied in triplicate to each soil type. Fertilizers were applied as per manufacturer’s instructions. All treatments were added with the same amount of water. Ingredients are listed based on package labelling. Numbers indicate % w/w.

* organic matter 61.5%, organic carbon 35.7%

** water added to increase VSW of soil by 20%

### Detection of *B*. *pseudomallei* in soil samples

Soil DNA extraction was done as previously described [15,21]. Briefly, 20 g of soil were incubated with 20 mL of Ashdown’s broth for 39 hours shaking at 37°C, the soil supernatant was centrifuged twice and the pellet processed using the PowerSoil Kit (MoBio Laboratories, USA). Modifications included the addition of 0.8 mg of aurintricarboxylic acid (ATA) and 20 μL of proteinase K (20 mg / mL).

*B*. *pseudomallei* DNA was targeted by the well validated *B*. *pseudomallei* specific Type Three Secretion System-1 TTS1 real-time PCR [24,25].

For the microcosm experiment, DNA was extracted from 20 g of soil using the previously described semi-quantification method with an internal extraction and amplification plasmid control [23]. TTS1 copy numbers were normalized by dividing them by the copy number of the internal pt7 plasmid control which was added to the soil samples prior to extraction, in order to account for differences in DNA extraction and amplification efficiency as a result of varying amounts of inhibitors present in soil samples [23].

### Statistical analysis

Statistical analysis was carried out using Stata (Intercooled Stata, version 12.1, USA). For bivariate analyses, Fisher’s exact test and Mann-Whitney U test were used. All tests were 2-tailed and considered significant if P values were less than 0.05. Graphs were generated in Stata and GraphPad Prism 6.

For the experimental field site, a conditional logistic regression model was used to model the odds of *B*. *pseudomallei* occurrence once the experiment had started, with fixed effects for treatments and dates of sampling. Fractional treatment effects were assumed for the first 12 months (e.g. 50% of full treatment effect after 6 months) to allow the application of fertilizers to have a gradual effect on the soil environment and *Burkholderia* community [26].

Heat maps for the experimental field site were generated using the thin-plate-spline interpolation method in ArcGIS 10.1 (ESRI 2012).

## Results

### The experimental field site

*B*. *pseudomallei* occurrence was monitored over three years on a field site with five different treatments applied in an area in rural Darwin naturally positive for *B*. *pseudomallei* (Fig. 1)

#### *B. pseudomallei* and the treatments

**Fig 2:**
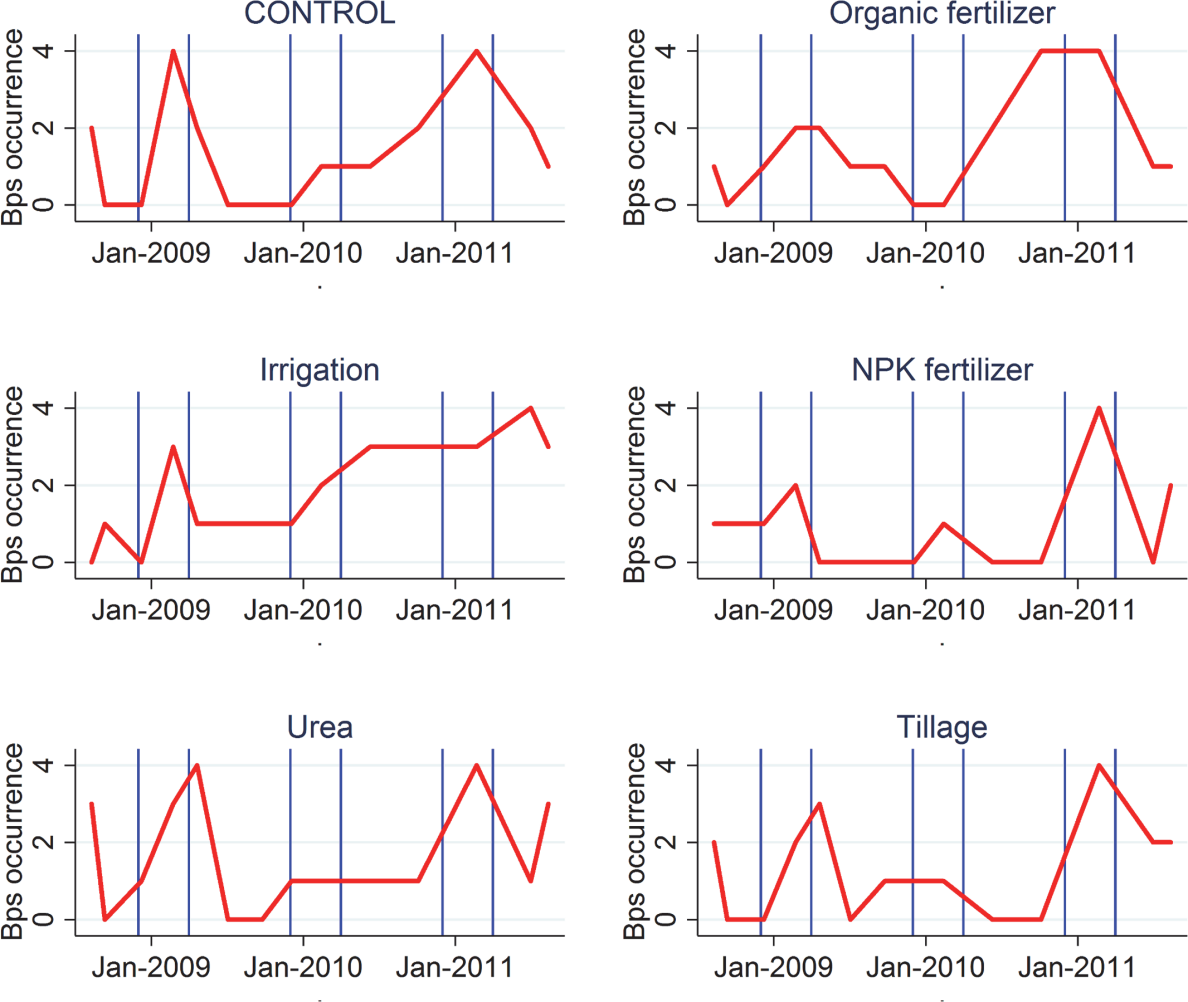
Longitudinal occurrence of *B*. *pseudomallei* (red line) on the experimental field site. The vertical blue lines indicate the start and end of the wet seasons. The y axis depicts the *B*. *pseudomallei* occurrence at total four holes per duplicate quadrants. The first measure was taken before treatments were applied.

**Fig 3:**
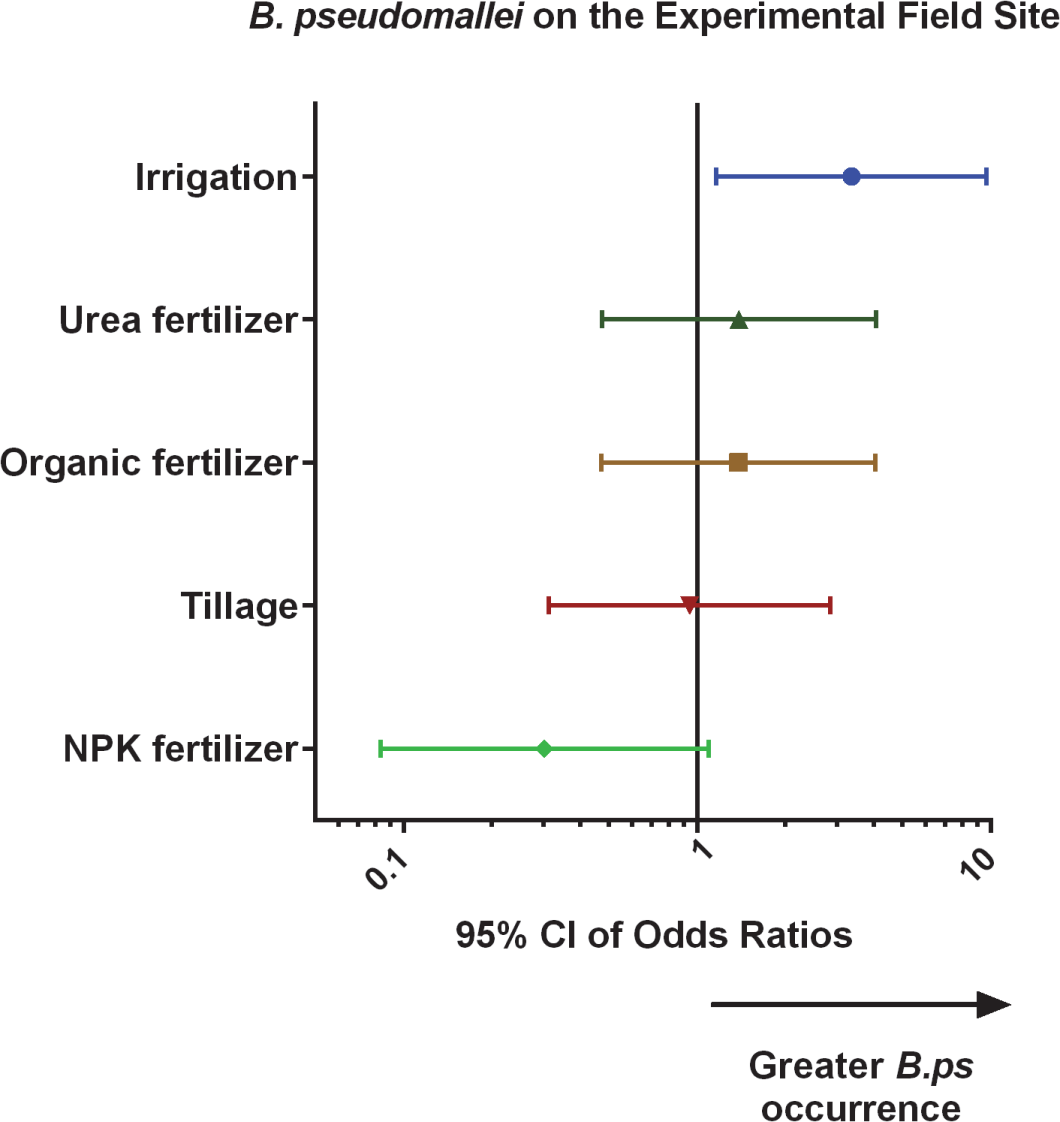
Results of the analysis on the occurrence of *B*. *pseudomallei* on the experimental field site. The odds ratio refers to the ratio of the odds of *B*. *pseudomallei* occurring in soil samples taken a year or more into the experiment from the treatment quadrants to the odds of *B*. *pseudomallei* occurring in soil samples from the control quadrants.

Of the 336 soil samples taken, 118 (35%) contained *B*. *pseudomallei*. The breakdown by quadrant is shown in Fig. 1. The breakdown by treatment and time is shown in Fig. 2. Irrigation was found to be the only treatment where the occurrence of *B*. *pseudomallei* during the experiment was significantly higher than that in the control quadrants (Fig. 3). Soil samples from irrigated quadrants had 3.3 times higher odds of containing *B*. *pseudomallei* compared to soil samples from the control quadrants (P = 0.025). Similar results were obtained when baseline *B*. *pseudomallei* for a quadrant was added to the model.

#### *B. pseudomallei* and physicochemical parameters

**Fig 4:**
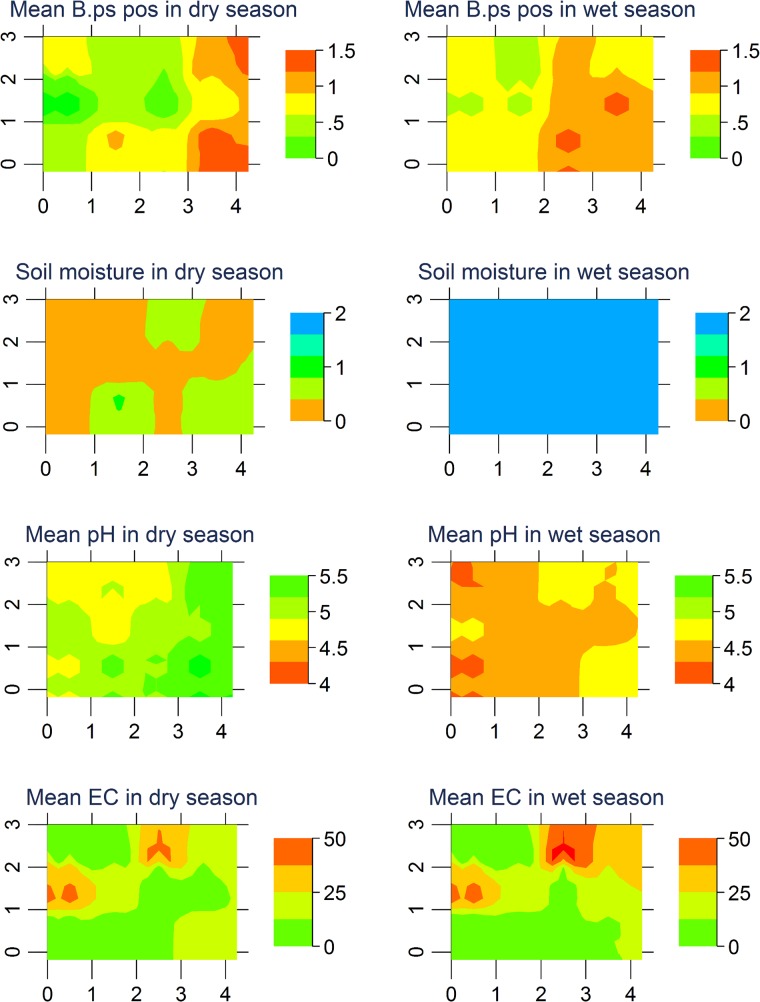
Heat maps for the mean *B*. *pseudomallei* occurrence per quadrant for the dry and wet season; mean soil moisture (“0” dry (<4% vsw), “1” moist (4–20% vsw), “2” wet (>20% vsw)), mean pH and mean electrical conductivity EC, i.e. soil salinity (μS/cm).

A graphic comparison between *B*. *pseudomallei* positive quadrants, soil water status and pH indicated an association between *B*. *pseudomallei* positive areas, irrigated areas in the dry season, and more neutral soil pH (Fig. 4). In a multiple linear regression analysis with dates and treatments as explanatory variables, the soil pH on irrigation plots a year into the experiment was higher than on control plots by 0.3 on average (P = 0.029) (S1 Fig).

EC was measured for soils collected in the last six months of the experiment. NPK and organic fertilizer quadrants showed highest EC values with a mean of 39 and 23 μS/cm as compared to 11 for urea, 11 for irrigation, 8 for tillage, and 8 μS/cm for the control quadrants (Fig. 4 and S1 Fig). There was a weak, non-significant trend for *B*. *pseudomallei* to be associated with a lower EC (*B*. *pseudomallei* positive samples: median 13 μS/cm, interquartile range 8–27 μS/cm; negative samples: 21 and 9–37 μS/cm). Despite potential mixing of treatments across quadrants particularly in the wet season, the EC data suggested only a minimal exchange of salts and nutrients between quadrants as EC correlated with type of treatments and differed between neighbouring quadrants (Fig. 4).

#### *B. pseudomallei* and the vegetation

The vegetation on the field before the experiment started was a native *Sorghum* spp. (spear grass). During year-1 of the experiment, the exotic weed *Pennisetum pedicellatum* (annual “mission grass”) which is widespread in rural Darwin started to spread on the field (S1 Fig). While there was no significant association between mission grass and a specific treatment type, there was a trend for more mission grass to occur in the organic and NPK treated quadrants (S1 Fig). There was evidence for an association of *B*. *pseudomallei* with mission grass with 85% (93/109) of *B*. *pseudomallei* positive samples being from mission grass infested sites as compared to 64% (130/203) of negative samples (Fisher’s Exact P<0.001). A multivariable logistic model with the outcome being *B*. *pseudomallei* occurrence, and the covariates being soil pH, categorical soil moisture and mission grass showed that the presence of mission grass increased the odds of *B*. *pseudomallei* presence 2.2 times (P = 0.016). A unit increase in soil pH was associated with an OR = 2.1 reflecting the baseline acidic conditions (P<0.001). Soil saturated with water increased the odds 9.4 times (P<0.001). Water saturated soil was encountered during the sampling rounds in February, i.e. the peak of the wet season (S1 Fig).

### Soil microcosm experiment

The microcosm experiment was used to determine whether commercial fertilizers commonly used in gardens in the Darwin region increased *B*. *pseudomallei* load in soil. Eight different treatments of garden fertilizers were applied to each of four different soil types in triplicate. There were also triplicate water controls with only distilled water added and triplicate controls with nothing added.

**Fig 5:**
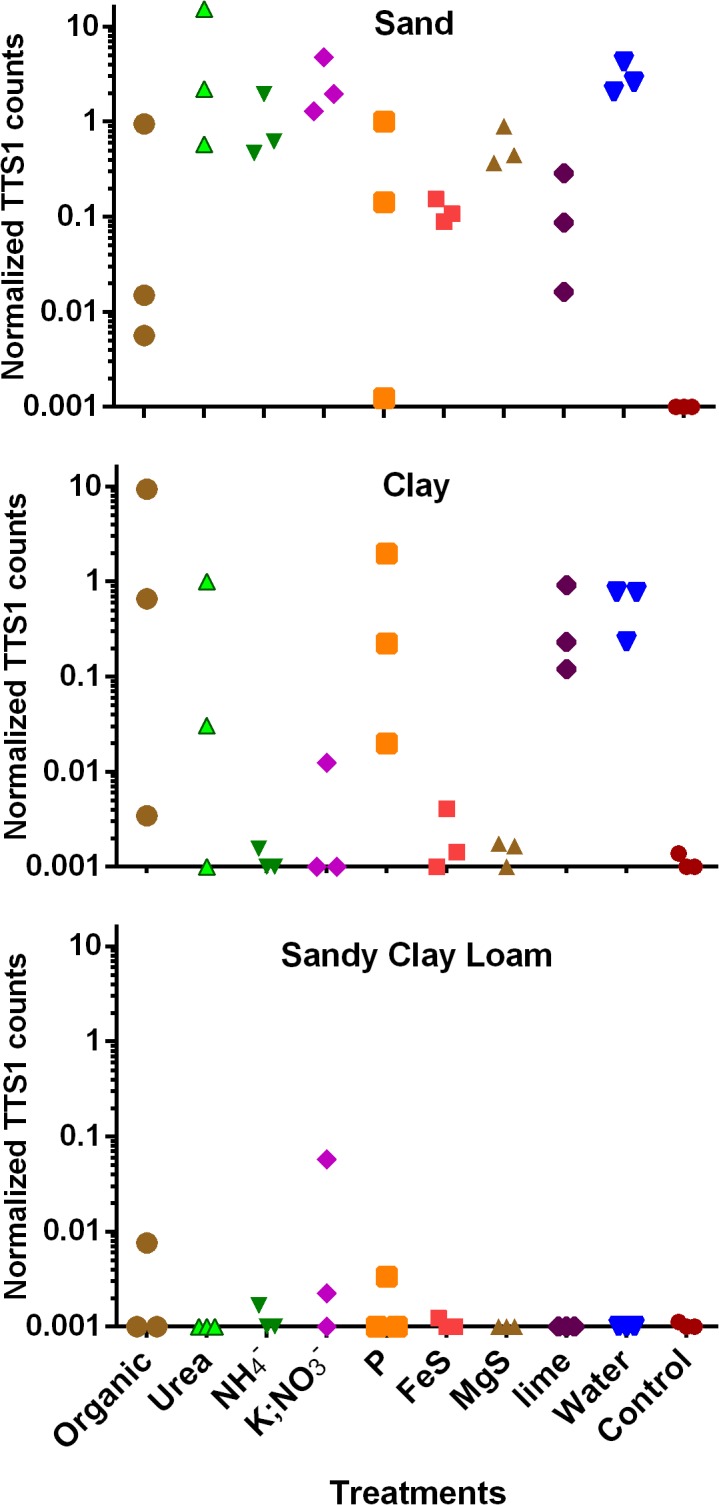
Microcosm experiment. *B*. *pseudomallei* load after 4 weeks inoculated in sand, sandy clay loam or clay with treatments in triplicate consisting of eight different fertilizers (Tables 2 and 3), a water-only treatment and a control with no change. The y-axes are in log scale (+0.001) and represent the normalized *B*. *pseudomallei* load with the ratio of TTS1 copy number over pt7 control plasmid numbers.

Four weeks after inoculation, no *B*. *pseudomallei* were detected in commercial garden soil for any of the treatments. Sand and clay contained on average 733 times more *B*. *pseudomallei* than sandy clay loam which only contained minimal *B*. *pseudomallei* cells (Fig. 5). After four weeks, no *B*. *pseudomallei* (10/12) or only minimal *B*. *pseudomallei* cells (2/12) were detected in the 12 control samples. The effect of fertilizers upon *B*. *pseudomallei* differed between soil type (Fig. 5). In sand, the addition of a fertilizer rich in urea showed the highest *B*. *pseudomallei* load compared to controls but the same fertilizer only had a moderate effect in clay and no effect in sandy clay loam. The pH and EC of urea in sand were lower with a mean of 4.6 and 56 μS/cm in comparison to sandy clay loam (pH 5.8 and EC 216 μS/cm) and clay (8.4 and 172 μS/cm). The addition of an organic and a phosphorus rich fertilizer resulted in the two highest mean *B*. *pseudomallei* counts in clay but no such effect was seen for the other soils. The addition of water alone caused a similarly high increase in *B*. *pseudomallei* load in sand and clay which had a low VSW of 0–2% before addition of water but there was no load increase in sandy clay loam with a higher initial VSW of 5%.

#### EC, pH and *B. pseudomallei*

There was good evidence for a negative association between *B*. *pseudomallei* load and soil EC when all soil types were combined (Spearman’s r = -0.29, P = 0.002). Soil EC was higher in soils with organic fertilizer, fertilizer rich in phosphorus, ammonium, urea or nitrates (median EC 147 μS/cm) when compared to soils with only water added (median EC 14 μS/cm; Mann-Whitney U test P<0.01 for all comparisons mentioned). Furthermore, no *B*. *pseudomallei* DNA was recovered from commercial composted organic-based “garden soil”, which had a VSW of 58%, a pH of 5.4 and a comparatively high EC of 700 μS/cm.

No association was found between soil pH and *B*. *pseudomallei* load with a pH range of 5.2 for the soils with iron and magnesium sulphates added to 8.0 for the soils containing garden lime. There was no significant reduction in *B*. *pseudomallei* load in soils with garden lime added.

## Discussion

Domestic gardens have been known for many years to be a source of acquisition of melioidosis [3]. It is not clear why this is the case other than gardens being a common meeting point between humans and the environment. However, our previous work has found an increased occurrence of *B*. *pseudomallei* in gardens in comparison to control areas in the dry season [15]. One of the main anthropogenic manipulations in gardens in the Darwin region is irrigation, i.e. regular watering during the prolonged mid-year dry season. Irrigation indeed proved to be the only treatment in this study to be associated with a significant longitudinal increase in occurrence of *B*. *pseudomallei* on the experimental field site. Furthermore, the addition of water was one of the main predictors for higher *B*. *pseudomallei* load in the microcosm experiment. This matches with previous reports that *B*. *pseudomallei* is often found on irrigated sports grounds [27,28] and golf courses; and irrigated rice fields are a known major risk factor in acquiring melioidosis in Southeast Asia [6,7,19].

*B*. *pseudomallei* occurrence generally increased across the field in 2010 which coincided with above average rainfall in the wet season 2009/2010 and high melioidosis case numbers during that time [2].

In addition to irrigation, the use of fertilizers is another common soil disturbance factor in domestic gardens. *B*. *pseudomallei* belongs to the Betaproteobacteria, a class with members including *B*. *pseudomallei* capable of ammonium oxidation, denitrification and polyphosphate accumulation, thereby providing a selective advantage over other bacteria in fertilized, eutrophic ecosystems [29]. A greater relative abundance of Betaproteobacteria was found in sediments of eutrophic reservoirs and agricultural wetland soils and abundance decreased after restoration [29]. In another study, a shift to *Burkholderia* spp. was evident after a change from forest to pasture vegetation [30] and tillage and fertilization have been shown to affect the *Burkholderia* community structure [26].

We found the impact of fertilizers upon *B*. *pseudomallei* to be complex and dependent on soil type, physicochemical soil parameters such as pH or salinity as well as biotic factors such as vegetation. In the microcosm experiment, a fertilizer rich in phosphorus or phosphates caused the highest mean *B*. *pseudomallei* load increase in clay with a neutral soil pH but only a small effect in sandy clay loam and sand. Phosphates adsorb to clay minerals due to the clay’s electrostatic surface and depending on soil pH, react with soil cations such as iron cations, making phosphates unavailable to microbes [31]. A neutral pH is the ideal range for maximum phosphate availability. Apart from essential functions of phosphates, *B*. *pseudomallei* uses phosphates to generate polyphosphates for oxidative stress response, motility and biofilm formation [32].

In the microcosm experiment, a fertilizer rich in nitrates increased *B*. *pseudomallei* growth across different soil types. These results match with a study conducted in Thailand, where *B*. *pseudomallei* was associated with soil containing more total nitrogen [9]. Nitrate is one of the biologically most important compounds in the nitrogen cycle and highly susceptible to leaching, thereby contaminating groundwater [33,34]. As a denitrifier *B*. *pseudomallei* reduces nitrates to nitrites as electron acceptors for anaerobic respiration [33]. Another nitrogen containing compound which increased *B*. *pseudomallei* growth in the microcosm experiment was a fertilizer rich in urea. Urea is hydrolysed to ammonia which is used by *B*. *pseudomallei* in biosynthetic pathways and is also oxidized to nitrates by nitrifying soil bacteria [35]. In previous studies *B*. *pseudomallei* occurrence was higher in areas where animals were kept (mainly horses and chickens) and the soil in these areas likely contained increased levels of urea [15,21].

However, neither urea nor the ammonium containing NPK fertilizer increased *B*. *pseudomallei* load on the fertilized field site. The latter might be due to the nutrient salts of the NPK fertilizer considerably increasing the soil salinity on these quadrants.

*B*. *pseudomallei* is a saprophyte so it was surprising not to find an association with the organic fertilizer in the field experiment. Concentrated organic material such as found in commercial potting mix has high buffering and cation exchange capacities, resulting in increasing pH and salinity. Indeed, no *B*. *pseudomallei* was recovered after four weeks in the tested commercial potting mix which showed exceptionally high EC values of up to 1,000 μS/cm. A preference of *B*. *pseudomallei* for less saline conditions and thus, less osmotic stress has previously been reported for *B*. *pseudomallei* in water, media and a soil microcosm study [17,36–38].

Nitrogen containing fertilizers are also known to acidify the soil in the long term with the release of hydrogen ions through nitrification processes by soil bacteria oxidizing ammonium to nitrites and nitrates [39]. Soil pH controls the availability of many nutrients in soil and is one of the strongest drivers of the soil bacterial community structure [29,40,41]. For *B*. *pseudomallei*, soil pH has previously been found to be an important abiotic soil parameter [9,15,17,36,37,42]. This study confirmed the preference of *B*. *pseudomallei* for a slightly more acidic soil but also with a decline in *B*. *pseudomallei* occurrence for pH below 5 [42,43]. The preference of *B*. *pseudomallei* for a more acidic soil makes it well equipped to grow in the weathered, lateritic soil commonly seen in tropical Australia. Interestingly a similar soil environment in Gabon has recently been shown to harbor *B*. *pseudomallei* [44]. This unmasking of the potential for endemic melioidosis in central Africa has important implications for ongoing studies that are attempting to define the geographical boundaries of the environmental presence of *B*. *pseudomallei* globally [10]. High annual monsoonal rainfall leads to excessive leaching with a depletion of alkaline cations in the topsoil, leaving behind hydrogen ions which contributes to the acidity of the soil as well as its low buffering capacity [41]. The pH on the experimental field site was indeed highly acidic to start with, having a median pH of 4.5 at baseline. After 3 years, the pH on the quadrants which were irrigated every 2^nd^ day rose to 6, most likely as a result of high ion load such as naturally occurring magnesium or calcium in the irrigation water, which was untreated bore water containing 50 mg/L calcium carbonate and with a pH of 7.5 [45]. The application of urea every two weeks also increased the pH from 4.5 to 5.5 due to urea hydrolysis releasing ammonia which was converted to ammonium at low soil pH.

No inhibitory effect of garden lime (32% w/w calcium carbonate) against *B*. *pseudomallei* was observed in the microcosm study after application as per manufacturer’s instructions (1% w/w). This matches a previous finding that even with quicklime (calcium oxide) which is more caustic than garden lime, a bactericidal effect against *B*. *pseudomallei* was only observed if mixed into the soil at considerable 40% w/w leading to a pH increase above 10 [46].

As a common habitat for bacteria of the *Burkholderia* genus, *B*. *pseudomallei* colonizes the rhizosphere and aerial parts of various plants such as grasses of the family *Poaceae* [23,47,48]. In particular exotic grasses introduced to Australia for pasture such as *Brachiaria humidicola* and *Pennisetum pedicellatum* (annual “mission grass”) have been found to be colonized by *B*. *pseudomallei* [23]. On the experimental field site, mission grass started to appear during the first year of the experiment, replacing native *Sorghum* spp. The presence of mission grass was a significant predictor for the presence of *B*. *pseudomallei* in a multivariable model accounting for soil pH and moisture, supporting previous findings of *B*. *pseudomallei* colonizing these grasses. These results suggest that while mission grass might have influenced the occurrence of *B*. *pseudomallei* across the field; there was no evidence that this grass preferentially occurred on the irrigated quadrants and thus, the growth of mission grass could not explain the association between *B*. *pseudomallei* and irrigation.

Statistical power was limited with four replicates per treatment per time point for 13 time points on the experimental field site, and three replicates per treatment per soil type in the microcosm experiment. Further studies are recommended to confirm the results. Furthermore, a small amount of mixing of treatments across quadrants of the experimental field could not be excluded; however, salinity data indicated no or only minimal mixing. Remediation measures to decrease *B*. *pseudomallei* load in gardens also need more formal study. Measures might include a reduction of irrigation and improved drainage as well as increasing the buffering capacity of the soil causing a rise in soil pH and salinity [46]. While a large amount of quick lime is needed to raise the soil pH and ultimately decrease *B*. *pseudomallei* counts [46], the use of potting mix might help increase the soil salinity due to its high cation exchange capacity. A reduction of fertilizers such as those containing nitrates might also assist in reducing load as well as restoration of native vegetation, with the latter also requiring less irrigation. It was previously reported that at a location in Western Australia *B*. *pseudomallei* was no longer detected after removal of chemical fertilizers and restoration of native vegetation [49].

### Conclusions

In summary there was clear evidence for irrigation increasing *B*. *pseudomallei* occurrence. The effect of fertilizer application upon *B*. *pseudomallei* was more complex and was dependant on soil type and physicochemical properties as well as on vegetation, with nutrients also causing an increase in plant root development beneficial to *B*. *pseudomallei*. The use of fertilizers is causing drastic changes to the global nutrient cycle with a significant rise in supply of otherwise limiting nutrients. These changes have a major impact upon the soil and water microbial community structure and likely also upon host pathogen interactions [50], including those involving *B*. *pseudomallei*.

## Supporting Information

S1 Fig Mean pH (A), soil moisture (B), electrical conductivity EC (C) and occurrence of mission grass (D) of the 4 replicates at a time point on the experimental field site. The vertical grey lines indicate the start and end of the wet seasons (Nov to April).
(DOCX)
