# Supplementary material for: What Drives the Occurrence of the Melioidosis Bacterium Burkholderia pseudomallei in Domestic Gardens?
Source: PLoS Negl Trop Dis. 2015 Mar 24;9(3):e0003635. doi: 10.1371/journal.pntd.0003635 (PMC4372393; doi:10.1371/journal.pntd.0003635)
Supplement: S1 Fig — The vertical grey lines indicate the start and end of the wet seasons (Nov to April). (DOCX) [file pntd.0003635.s001.docx]

**Supporting Information**

**S1 Fig. Mean pH (A), soil moisture (B), electrical conductivity EC (C) and occurrence of mission grass (D) of the 4 replicates at a time point on the experimental field site.** The vertical grey lines indicate the start and end of the wet seasons (Nov to April).

**A)**

**B)**

**C)**

**D)**
